# Inhibition of NOS1 promotes the interferon response of melanoma cells

**DOI:** 10.1186/s12967-022-03403-w

**Published:** 2022-05-10

**Authors:** Xi Chen, Zhiwei Zou, Qianli Wang, Wenwen Gao, Sisi Zeng, Shuangyan Ye, Pengfei Xu, Mengqiu Huang, Keyi Li, Jianping Chen, Zhuo Zhong, Qianbing Zhang, Bingtao Hao, Qiuzhen Liu

**Affiliations:** 1 Cancer Research Institute, Experimental Education/Administration Center, School of Basic Medical Science, Southern Medical University, Shatai South Road, Baiyun District, 16, Guangzhou, 510515 China; 2 Pingshan District People’s Hospital of Shenzhen, Shenzhen, 518118 China; 3 First Affiliated Hospital of Zhengzhou University, Zhengzhou University, Zhengzhou, 450001 China; 4 Guangzhou Hospital of integrated Traditional and West Medicine, Guangzhou, 510800 China

**Keywords:** CRISPR/Cas9, Bioinformatics, NOS1, Metabolism, Interferon, Melanoma

## Abstract

**Background:**

NOS1 expression predicts poor prognosis in patients with melanoma. However, the molecular function of NOS1 in the type I IFN response and immune escape of melanoma is still unknown.

**Methods:**

The CRISPR/Cas9 system was used to generate NOS1-knockout melanoma cells and the biological characteristics of NOS1-knockout cells were evaluated by MTT assay, clonogenic assay, EdU assay, and flow cytometric assay. The effect on tumor growth was tested in BALB/c-nu and C57BL/6 mouse models. The gene expression profiles were detected with Affymetrix microarray and RNA-seq and KEGG (Kyoto Encyclopedia of Genes and Genomes) and CLUE GO analysis was done. The clinical data and transcriptional profiles of melanoma patients from the public database TCGA (The Cancer Genome Atlas) and GEO (Gene Expression Omnibus, GSE32611) were analyzed by Qlucore Omics Explorer.

**Results:**

NOS1 deletion suppressed the proliferation of melanoma A375 cells in culture, blocked cell cycling at the G0/G1 phase, and decreased the tumor growth in lung metastasis nodes in a B16 melanoma xenograft mouse model. Moreover, NOS1 knockout increased the infiltration of CD3+ immune cells in tumors. The transcriptomics analysis identified 2203 differential expression genes (DEGs) after NOS1 deletion. These DEGs indicated that NOS1 deletion downregulated mostly metabolic functions but upregulated immune response pathways. After inhibiting with NOS1 inhibitor N-PLA, melanoma cells significantly increased the response to IFN$\upalpha$ by upregulation expression of IFN$\upalpha$ simulation genes (ISGs), especially the components in innate immune signaling, JAK-STAT, and TOLL-LIKE pathway. Furthermore, these NOS1-regulating immune genes (NOS1-ISGs) worked as a signature to predict poor overall survival and lower response to chemotherapy in melanoma patients.

**Conclusion:**

These findings provided a transcriptional evidence of NOS1 promotion on tumor growth, which is correlated with metabolic regulation and immune escape in melanoma cells.

**Supplementary Information:**

The online version contains supplementary material available at 10.1186/s12967-022-03403-w.

## Background

Endogenous nitric oxide (NO) produced by three isoforms of nitric oxide synthase (NOS1, NOS2 and NOS3) is involved in a broad spectrum of physiological and pathological activities such as immune responses, neurotransmission, and smooth muscle relaxation [1, 2]. NOS genes are abnormally up-regulated in various cancers and the increased expression is correlated with poor survival [3, 4]. NOSs regulate multiple biological functions including autophagy, metabolism and proliferation by producing a low level of nitric oxide in tumors [5–7]. Nitric oxide produced by NOSs exerts dual functions in a cGMP-dependent or cGMP-independent manner [8–10]. In the cGMP-dependent manner, nitric oxide reacts with the active site of soluble guanylate cyclase, leading to enzymatic activation and subsequent activation of MAPK cascade [11, 12]. The cGMP-independent pathway is commonly through modification of target proteins [9, 13]. NO-nitrosylated cysteine residues of proteins altered functional properties of energy metabolism such as mitochondrial respiration/proliferation and reactions of glucose metabolism [13, 14]. NO can react with superoxide anions to yield peroxynitrite, which nitrates with amino acids to form nitrotyrosine (nitration) and leads to the downregulation of the JAK/STAT pathway, contributing to IFN$\upalpha$ tolerance of tumor cells [15].

More and more studies indicated that NOS2 expressed in tumor-infiltrating immune cells such tumor associated macrophages (TAMs) and myeloid-derived suppressor cells (MDSCs) play a critical role in cancer immune modulation [5, 16]. NOS2-derived NO inhibits the proliferation of effector T cells and downregulation of IFN$\upgamma$ productions, but promotes the differentiation of regulatory T cells in tumor microenvironment [17]. The expression and function of the three isoforms of NOS differ in different cell types [4]. NOS1 is a calcium-dependent, constitutively expressed nitric oxide synthase that can produce low levels of nitric oxide in a variety of tissues and tumors [17]. Unlike the other two isoforms, NOS1 interacts with target proteins through its unique PDZ domain, which ensures the effectiveness and selectivity of the NOS1 function [18, 19]. Our study showed that NOS1 bound with the key enzyme of glycolysis and modulated metabolic rewiring in ovary cancer progression [20]. In melanoma, we found NOS1 expression correlated negatively with phosphorylation of STAT1 in IFN$\upalpha$ signal of patients PBMCs and predicted poor response to adoptive T cell therapy [21]. Recently, we found that NOS1 inhibited the IFN$\upalpha$ response through binding and S-nitrolating histone deacetylase HDAC2 [22].

Here, we generated NOS1-knockout melanoma cells to explore the role of NOS1 on melanoma growth and interferon response, and provide possible mechanisms through global transcription analysis.

## Methods

### Cell culture

Human melanoma A375 cell line and murine melanoma B16 cell line were maintained in our laboratory. Cells were cultured in RPMI-1640 medium (Gibco, Gaithersburg, MD, USA) supplemented with 10% fetal bovine serum (BI, Salt Lake City, UT, USA), and maintained at $37\;^{\circ }\hbox {C}$ in a humidified atmosphere of 5% CO2.

### Plasmid construction

CRISPR/Cas9 Plasmid p2u6 was approved by Zhili Rong, Ph.D. [23] The gRNAs were designed using the online software (http://crispr.mit.edu). The gRNAs were amplified by PCR using Q5 High-Fidelity DNA Polymerase (NEB). To construct plasmids, PCR products and Plasmid p2u6 linearized by BaeI digestion were used for Gibson assembly (NEBuilder HiFi DNA Assembly Master Mix). The sequence of gRNAs is listed in Table 1.

**Table 1:** Sequences of the single guide RNAs (gRNAs)

gRNA name	Sequence (5’–3’)
gRNA1	CAGCGCGAGGCATTCCGCC
gRNA2	CTATCAGATGATGGTCACG

### DNA extraction and genotyping

The genomic DNA was extracted from the cell using an SDS lysis buffer. The genomic DNA was subjected to PCR amplification to identify the mutations. The mutation was confirmed by sequencing the PCR product, and the primers used are listed in Table 2.

**Table 2:** Sequence of the primers used for genotyping

Primer name	Forward primer (5’–3’)	Reverse primer (5’–3’)
NOS1	CCCACATGGAAAGGCTGGAA	AGAGCTGGCTGTGATCTTGG

### Western blotting

Total protein was extracted by a Protein Extraction Kit (Fude, Hangzhou, CN). The concentration in each sample was determined using the Bradford assay (Beyotime, Shanghai, CN). Equal amounts of proteins were separated on 8–15% SDS-PAGE. Then the samples were transferred to PVDF membranes (Millipore, USA) and blocking with 5% BSA for 1h. Before corresponding with HRP-conjugated secondary antibodies, the membranes were incubated with the primary antibodies, anti-NOS1 (Abcam, Cambridge, MA, USA) at a 1:1000 dilution and GAPDH (Proteintech, Wuhan, CN) at a 1:5000 dilution, respectively. Then, the immunoreactive bands were visualized by chemiluminescence (Bio-Rad, USA).

### MTT assay

Cells were implanted into 96-well plates and cultured overnight. The next day, 10 $\upmu$l 5 mg/ml MTT (Sigma-Aldrich, St. Louis, USA) was added to each well. After incubated for 2–4 h at 37 $^{\circ }\hbox {C}$, 100$\upmu$l of DMSO was added to each well. Swirl gently for 15min at RT. After purple precipitate was fully dissolved, record absorbance at 490nm by BioTek Instruments (Winooski, VT, USA).

### Colony formation assay

Cells were implanted into 6-well plates. Fresh medium was added to plates every 3 days. On day 10, cells were stained with 0.005% crystal violet and photographed after fixed with 4% paraformaldehyde for 15min.

### EdU assay

Cells were implanted into 96-well plates and cultured overnight. The next day, $50 \; \upmu$M EdU was added to each well for 2h at $37 \; ^{\circ }\hbox {C}$. Subsequently, the cells were fixed with 4% paraformaldehyde for 30min. Then, incorporated EdU was detected using the Cell-Light EdU Apollo 567 in Vitro Kit (RiboBio, Guangzhou, CN).

### Flow cytometry cell cycle analysis

Cells were pelleted and washed with ice-cold PBS buffer, then incubated with PI staining solution at $4 \; ^{\circ }\hbox {C}$ for 30min. The cell-cycle distribution was determined by FACS Aria Flow cytometer (Beckton Dickson, San Jose, CA, USA). PI staining solution: 25mg PI + 25mg Sodium Citrate + 5 mg RNase A + 750 ml Triton X-100 in 250 ml ddH${}_2$O.

### Animal experiments

All animals in this study, C57BL/6 mice and BALB/c-nu mice (Female, 6weeks old), were all purchased from Guangdong Medical Laboratory Animal Center.

Tumor xenograft assay was performed as described below. Briefly, $5\times 10{}^6$ A375 cells (NOS1 KO or WT) were injected subcutaneously into BALB/c-nu mice($\hbox {n}=5$). Tumor size was measured by caliper every 3 days. And tumor volume was calculated according to the following equation: tumor volume (mm${}^3$) = (length (mm) $\times$ width${}^2$ (mm${}^2$)) $\times \; 0.5$. The xenograft experiment was terminated when tumors were no more than 1400 mm${}^3$ in volume.

To construct lung metastasis models of melanoma, $5\times 10{}^5$ B16 cells (NOS1 KO or WT) were intravenously injected into C57BL/6 mice ($\hbox {n}=7$). Mice were euthanized on day 11 post-injection, and lung tissue was isolated and photographed. Paraffin sections 5 ($\upmu \hbox {m}$) were stained with H&E, examined by microscopy and photographed.

### Real-time PCR

Total RNA was isolated using an RNAiso Plus reagent (Takara, Shiga, Japan) and reverse-transcribed using a PrimeScript RT kit (Takara). Then, TB Green Premix Ex Taq II (Takara) and LightCycler 96 System (Roche Life Science) were performed on quantitative real-time PCR (qPCR) analyses. Gene expressions were calculated using the comparative $2{}^{-\Delta \Delta CT}$ method. The primer sequences for qPCR are listed in Table 3.

**Table 3:** Sequence of the primers used for verifying JAK-STAT pathway

Gene name	Forward primer (5’–3’)	Reverse primer (5’–3’)
GAPDH	CTCCAAAATCAAGTGGGGCG	TGGTTCACACCCATGACGAA
IL13RA2	AAAGTTCAGGATATGGATTGCGT	GAAGTACACCTATGCCAGGTTTC
SPRY4	TCTGACCAACGGCTCTTAGAC	GTGCCATAGTTGACCAGAGTC
PIK3R3	TACAATACGGTGTGGAGTATGGA	TCATTGGCTTAGGTGGCTTTG
LIF	CCAACGTGACGGACTTCCC	TACACGACTATGCGGTACAGC
IFNAR2	TCATGGTGTATATCAGCCTCGT	AGTTGGTACAATGGAGTGGTTTT
CCND1	GCTGCGAAGTGGAAACCATC	CCTCCTTCTGCACACATTTGAA
IL24	CACACAGGCGGTTTCTGCTAT	TCCAACTGTTTGAATGCTCTCC
EPOR	ACCTTGTGGTATCTGACTCTGG	GAGTAGGGGCCATCGGATAAG
IRF9	GCCCTACAAGGTGTATCAGTTG	TGCTGTCGCTTTGATGGTACT
IFNGR2	CTCCTCAGCACCCGAAGATTC	GCCGTGAACCATTTACTGTCG
IFNAR1	AACAGGAGCGATGAGTCTGTC	TGCGAAATGGTGTAAATGAGTCA
JAK1	CTTTGCCCTGTATGACGAGAAC	ACCTCATCCGGTAGTGGAGC

### RNA extraction, ss-cDNA synthesis and microarray analysis

Total RNA was extracted by TRIzol (Invitrogen, CA) and purified with NucleoSpin miRNA (Macherey-Nagel, Duren, DE). Target preparation for microarray processing was carried out according to the GeneChip WT PLUS Reagent Kit. A total of 500 ng RNA was used for a double-round of cDNA synthesis. The cDNAs were fragmented, biotinylated, and hybridized to the Affymetrix Clariom S Assay human (Affymetrix, Santa Clara, CA). Following hybridization, the microarrays were washed and stained with Streptavidin Phycoerythrin on the Affymetrix Fluidics Station 450. Microarrays were scanned by using Affymetrix GeneChip Command Console (AGCC, Thermo Fisher Scientific, Inc) which installed in GeneChip Scanner 3000 7G. The microarray data are deposited in the GEO database with the accession GSE166287.

The data were analyzed with Robust Multichip Analysis (RMA) algorithm using default analysis settings and global scaling as normalization method Values presented are log2 RMA signal intensity. Normalized data were further analyzed using a moderated t-test to screen out the DEGs (differentially expressed genes) with Fold Change $>1.2$ or $<-1.2$, $\hbox {FDR}<0.05$. The volcano plot was constructed using the R studio web server iBio Tools v5.0. Kyoto Encyclopaedia of Genes and Genomes pathway enrichment analyses of DEGs were performed by the KOBAS online database(http://kobas.cbi.pku.edu.cn/kobas3). Cluego plug-in in Cytoscape software (3.8 version) was used for showing the ClueGO network diagram. GSEA software(https://www.gsea-msigdb.org/gsea/index.jsp) was used to perform the gene set enrichment analysis. A normalized enrichment score (NES) and p-value $<0.05$ were used to determine statistical significance.

### RNA extraction, library preparation, Illumina Hiseq xten/Nova seq 6000 Sequencing and RNA-seq data analysis

Total RNA was extracted using TRIzol (Invitrogen) and genomic DNA was removed using DNase I (TaKara). A total of $1 \;\upmu \hbox {g}$ mRNA was isolated following the TruSeq RNA sample preparation kit (Illumina, San Diego, CA). The double-stranded cDNA was synthesized using a SuperScript double-stranded cDNA synthesis kit (Invitrogen, CA). Then, the synthesized cDNA was degraded and selected. After quantified by TBS380, paired-end RNA-seq sequencing library was sequenced with the Illumina HiSeq xten/NovaSeq 6000 sequencer. The RNA-seq data are deposited in the SRA database with the accession PRJNA700578. The raw paired-end reads were analyzed by SeqPrep (https://github.com/jstjohn/SeqPrep) and Sickle (https://github.com/najoshi/sickle) with default parameters. Then clean reads were separately aligned using TopHat (http://tophat.cbcb.umd.edu/, version2.0.0) software.

DEGs were screened by R statistical package software EdgeR (Empirical Analysis of Digital Gene Expression in R, http://www.bioconductor.org/packages/2.12/bioc/html/edgeR.html) with Fold Change $>1.2$ or $<-1.2$, $\hbox {FDR}<0.05$. Kyoto Encyclopaedia of Genes and Genomes pathway enrichment analyses of DEGs were performed by the KOBAS online database(http://kobas.cbi.pku.edu.cn/kobas3).

Clinical data were all performed using the NCI BRB-Array tool [24] and Qlucore Omics Explorer 3.2 (https://www.genomeweb.com/resources/new-product/qlucore-omics-explorer-32).

### Statistical analysis

The Student’s t-test was used to analyze the two-sample comparison. A p-value $<0.05$ was considered to be statistically significant. The values are expressed as the mean values ±S.D. All the statistical analyses were performed using Prism software, version 8.0 (GraphPad, Inc, San Diego, CA, USA).

## Results

### NOS1 deletion suppresses the growth of melanoma cells

**Fig. 1:**
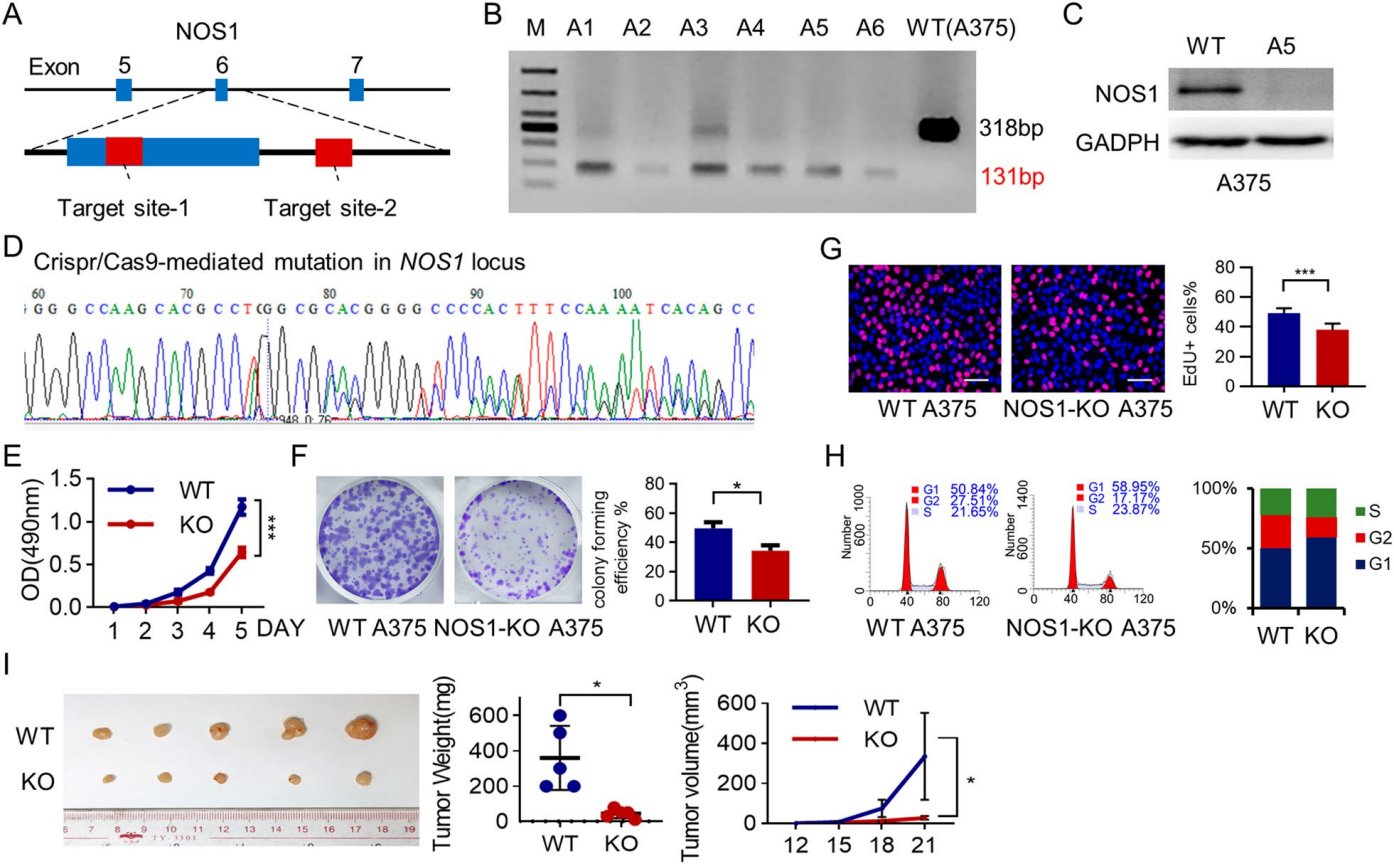
NOS1 is essential for cell proliferation and tumor growth of melanoma. ** A** Schematic diagram of NOS1-targeting guide RNA (gRNA) sequences. ** B** Knockout of NOS1 gene was confirmed by PCR assay with 131bp compare to 318bp in wide-type allele. A2, A4, A5 and A6 clone displayed NOS1 deletion. ** C** Western blot showed that NOS1 knockout in A5 clone. ** D** The sequence analysis confirmed A5 clone with 180bp deletion and 1bp mutation. ** E** MTT assay showed NOS1-KO cells grow slower than WT cells (***$\hbox {p}<0.001$). ** F** Plate formation assay indicated the clone number of NOS1-KO group was less than WT group. (*$\hbox {p} < 0.05$). ** G** The reprehensive image (left panel ) of EdU staining(red) for proliferation cells of NOS1-KO and WT cells , and the proliferation cells was decreased in NOS1-KO cells compared to WT cells (right panel) (Scale bars, $500 \; \upmu \hbox {m}$. ***$\hbox {p} < 0.001$). ** H** Flow cytometry assay (PI staining) showed cell cycle was blocked at G1 phase by NOS1-KO.** I** The tumors of NOS1-KO and WT A375 in BALB/c-nu mouse at the termination of experiment (n = 5, left panel). The tumor weight (middle panel) and tumor growth curves (right panel) in NOS1-KO group were lower than WT group (*$\hbox {p} < 0.05$)

To generate NOS1-deleted melanoma cell lines, we constructed a CRISPR/Cas9 plasmid with a gRNAs targeting the exon six of NOS1 gene (Fig. 1A and Additional file 1: Fig. S1) and transfected into A375 cells. Six clones with NOS1 mutated alleles were selected with puromycin and four clones (A2, A4, A5, and A6) were identified mutated successfully by PCR assay (Fig. 1B). Western blot assay confirmed that NOS1 expression was completely abolished in the A5 clone (Fig. 1C), and Sanger sequencing analysis showed a 180bp deletion and one site mutation at NOS1 alleles (Fig. 1D). Thus, we generated a NOS1-knockout melanoma cell line NOS1-KO A375 (clone A5) for the following experiments.

First, we detected the effect of NOS1 deletion on cell growth in culture using the MTT assay and clone formation assay. The data showed that the proliferation rate and the clonogenic ability of NOS1-KO A375 cells decreased by about 50% compared to WT cells (Fig. 1E, Fig. 1F, $\hbox {p}<0.001$, $\hbox {p}<0.05$). Harvesting the exponential growth cells and staining with EdU, we compared the number of EdU positive (EdU+) cells between NOS1-KO A375 cells with control cells. NOS1 deletion decreased the proliferation of A375 cells (Fig. 1G). Cell cycle analysis with PI staining showed that the G1, G2, and S-phase were 58.95%, 17.17%, and 23.87% in NOS1-KO cells and 50.84%, 27.51%, and 21.65% in WT A375 (Fig. 1H), indicating NOS1 knockout may reduce G1/S phase transition and prevent cell proliferation.

The xenograft mouse model experiment (NOS1-KO and WT A375 cells in BALB/c-nu mice, five mice for each genotype) showed that NOS1 deletion dramatically reduced the tumor sizes, which were measured by three diameters and tumor weights, ($\hbox {p} < 0.05$, Fig. 1I). These results suggested that NOS1 is essential for melanoma cell growth, especially for xenograft tumors.

### NOS1 deletion downregulated cancer-associated metabolic pathways

**Fig. 2:**
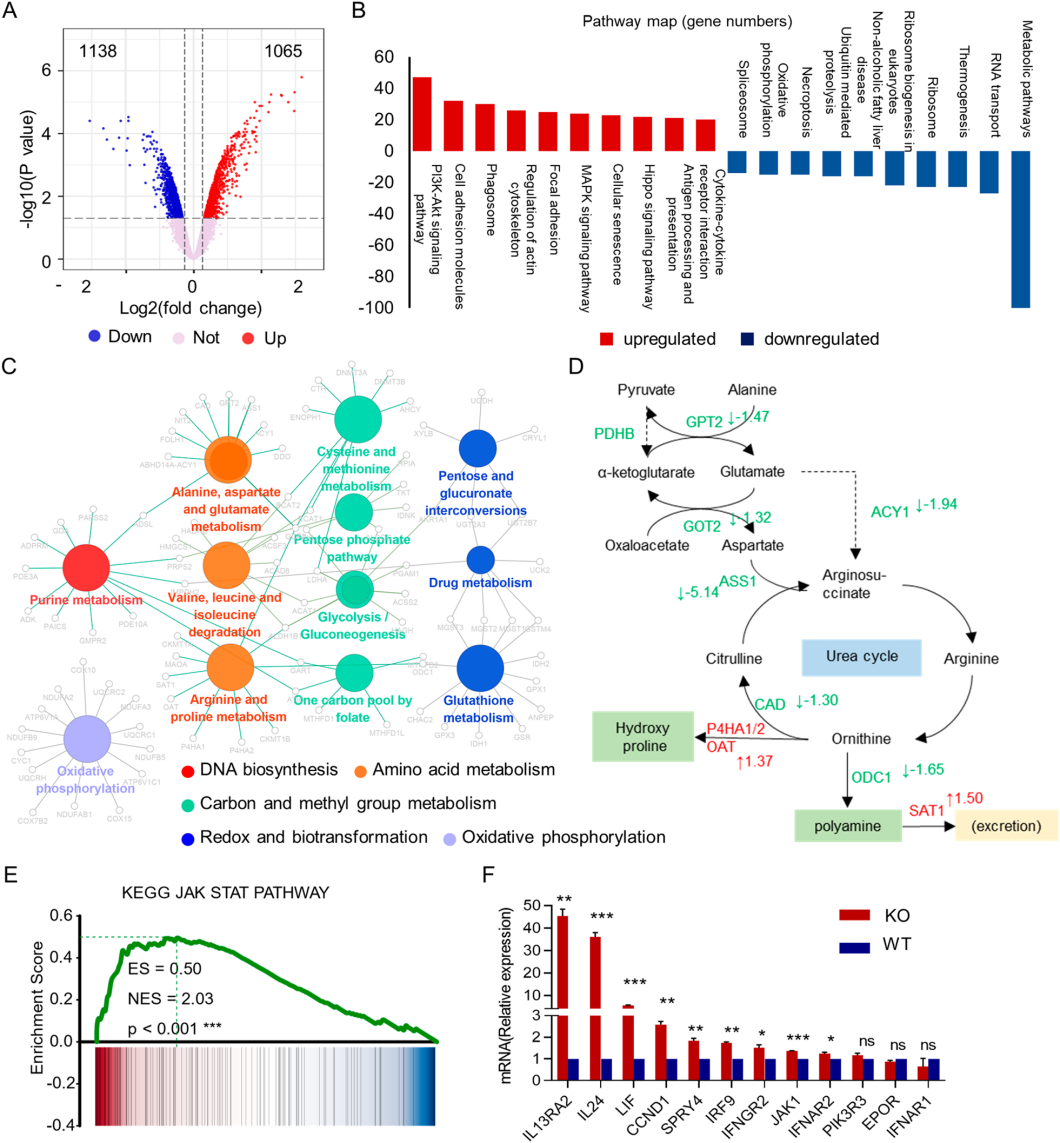
NOS1 deletion disturbed multiple metabolic pathways in melanoma cells. ** A** Volcano plot of the transcriptional data. X- axis: log2FC, Y-axis: –log10 (p-value). Red: upregulated gene, blue: downregulated gene. ** B** KEGG analysis displays the up and down pathways (top ten) of NOS1-KO differential expression genes. X-axis: the number of enriched gene, Y-axis: KEGG pathway. ** C** CLUE GO analysis indicated the metabolic pathways enriched genes are involved in macr omolecules biosynthesis. ** D** The schematic diagram of DEGs in urea cycle. Red: upregulated gene, Green: downregulated gene. ** E** GSEA showed JAK-STAT signaling pathway up-regulated by NOS1 deletion. ** F** qPCR validated the expression of 12 genes in JAK-STAT signal

We next explored the effect of NOS1 deletion on the transcription profile of melanoma A375 cells. Using Affymetrix microarray, we identified 2203 differentially expressed genes (DEGs) in NOS1-deleted cells, including 1138 downregulated and 1065 upregulated genes (cutoffs of $\hbox {p} < 0.05$ and $\hbox {FC} \ge 1.2$ or $\le 1.2$; Fig. 2A). KEGG analysis showed that the top ten pathways of downregulated DEGs are enriched in metabolic processes, while the top ten pathways of upregulated DEGs were involved in cancer signaling pathways cellular interaction, and focal adhesion (Fig. 2B).

The top one is metabolic pathways, which involved 164 genes. Network analysis using the CLUEGO of Cytoscape software showed that these metabolic genes were associated with twelve metabolic pathways, including purine metabolism, amino acid metabolism, carbon metabolism, and redox balance or biotransformation (Fig. 2C). Thus, transcription data and cell experiments in vitro indicated that NOS1 expression plays a role in cell cycle regulation of melanoma cells.

The transaminase GOT and GPT, and a key enzyme for the regeneration of arginine (ASS1) and CAD complex (carbamoyl-phosphate synthetase 2, aspartate transcarbamylase, and dihydroorotase) are downregulated in NOS1-deleted cells, implicating that NOS1 participates in ammonia metabolism and arginine regeneration. It has been reported that increased intracellular polyamine concentrations are essential for cell proliferation and tumorigenesis. Ornithine decarboxylase (ODC) is the rate-limiting enzyme for polyamine synthesis, and spermidine/spermine N-acetyltransferase (SAT1) a critical enzyme for polyamine exclusion, both of which are key regulators for intracellular polyamine concentration. We observed that the ODC expression decreased and the SAT1 level increased in NOS deleted cells, suggesting NOS1 plays a critical in regulating intracellular polyamine concentration (Fig. 2D).

Gene set enrichment analysis (GSEA) found that the gene expression of NOS1 deletion mainly enriched in 37 processes-related KEGG pathways (Additional file 2: Table S1). The up-regulation pathways (21 terms) mainly exhibited enrichment of immune disease and regulation, whereas the down regulation pathways (16 terms) showed enrichment of metabolism and cellular progresses. We noticed that the genes in JAK-STAT signaling pathway, a key signaling of innate immune regulation, significantly enriched in upregulated genes of NOS1 deletion (Fig. 2E). We tested the expression of components in JAK-STAT signal in NOS1-deleted cells using qPCR. Nine of 12 tested genes were upregulated in NOS1-deleted cells (Fig. 2F).

### NOS1 knockout activated the JAK-STAT signaling pathway

**Fig. 3:**
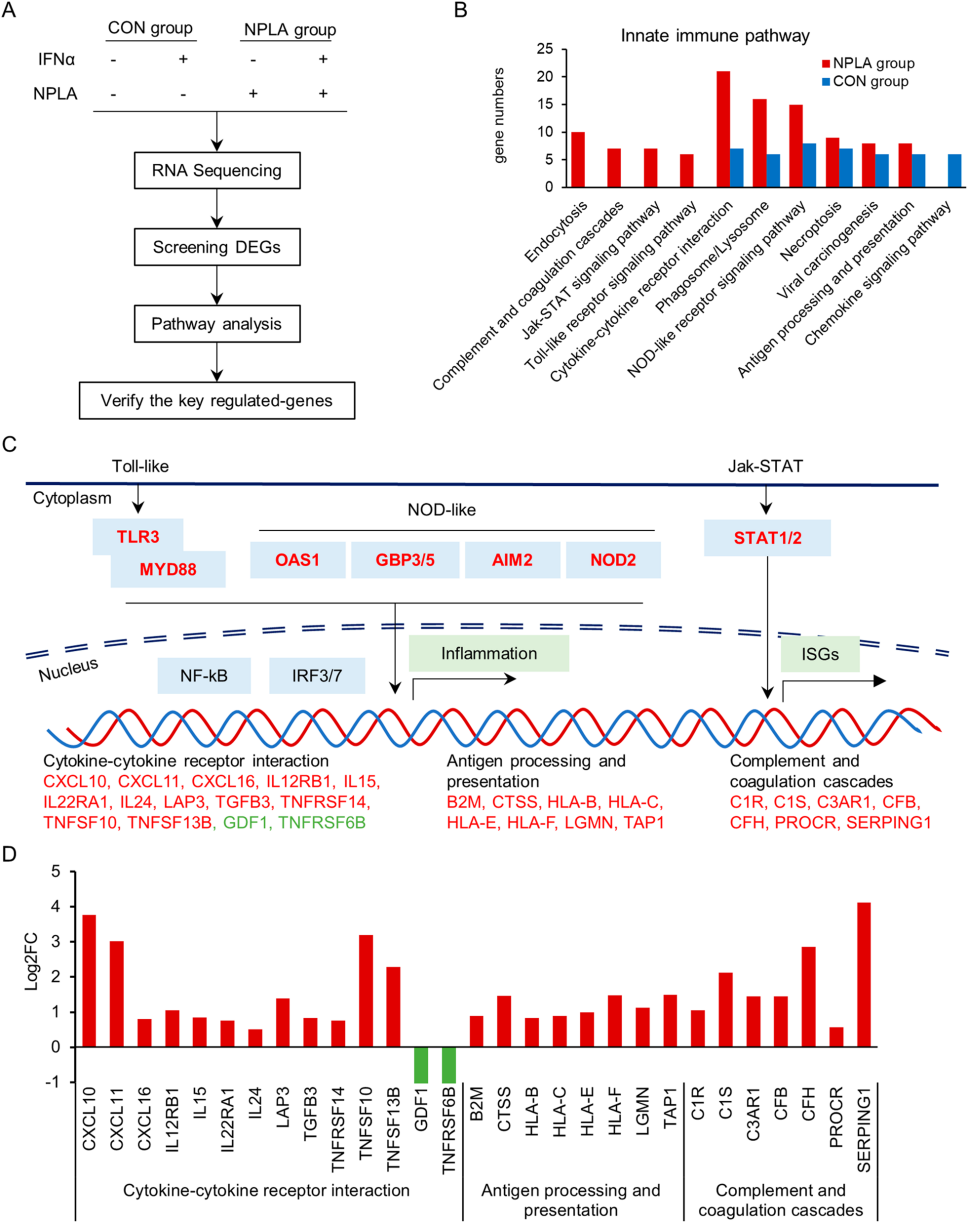
NOS1 regulated gene expression related to innate immune response. ** A** Schematic representation of the experimental design. ** B** KEGG analysis showed that the gene number and pathways enriched in interferon stimulating genes (ISGs) were increased by NOS1 inhibition (Red / Blue for NPLA / control group). ** C** Schematic diagram shows the up regulation of innate immune pathways in RNA-seq data with NOS1 inhibitor (N-PLA). The expression of key components of JAK-STAT and Toll-like pathway (TLR3, MYD88, STAT1/2, OAS1, AIM2, NOD2 and GBP3/5) and downstream signal events were increased by NOS1 inhibition. (Red and Green for up and down-regulation respectively). ** D** Log2FC of downstream genes were increased by NOS1 inhibition. (Red and Green for up and down-regulation respectively)

Dysfunction of JAK-STAT signal in cancer cells is crucial for cancer immune escape [21]. Therefore, we explored the role of NOS1 in the expression of genes in response to IFN$\upalpha$ (ISGs). A375 cells were stimulated with IFN$\upalpha$ (1000 IU/ml) for 12 hours with or without NOS1 inhibition ($100\upmu$M NPLA) and RNA-sequencing (RNA-Seq) was performed for gene expression profiling. The differential expression genes between cells with or without IFN$\upalpha$ stimulation were defined as ISGs. 290 ISGs were identified in A375 (CON-ISGs), while 460 ISGs identified in the NPLA-treatment group (NPLA -ISGs). Workflow of genomic comparison of NOS1-inhibitor versus NOS1-WT A375 cells was present in Fig. 3A.

KEGG analysis of ISGs indicated that NOS1 deletion induced upregulation of cytokine-receptor interactions and downstream signals of JAK-STAT signaling, TOLL-like receptor signaling, and NODs receptor signaling, but not chemokines signaling pathways (Fig. 3B). These data suggested the NOS1 may act mainly on pathways of the immune reaction regulation but not the chemotaxis of immune cells.

KEGG analysis showed that NOS1-ISGs enriched in immunoreaction processes, cytokine-receptor interactions, JAK-STAT signaling, TOLL-like receptor signaling, and NODs receptor signaling, antigen processing and presentation, complement and coagulation cascades (Fig. 3C, D). NOS1 inhibition increased expression of TLR3, MYD88, STAT1/2, OAS1, AIM2, NOD2 and GBP3/5, key factors in response to IFN$\upalpha$ stimulation. Thus, these data suggested that NOS1 is involved in innate immune escape of melanoma.

### NOS1 deletion inhibited melanoma growth in xenograft mouse model

**Fig. 4:**
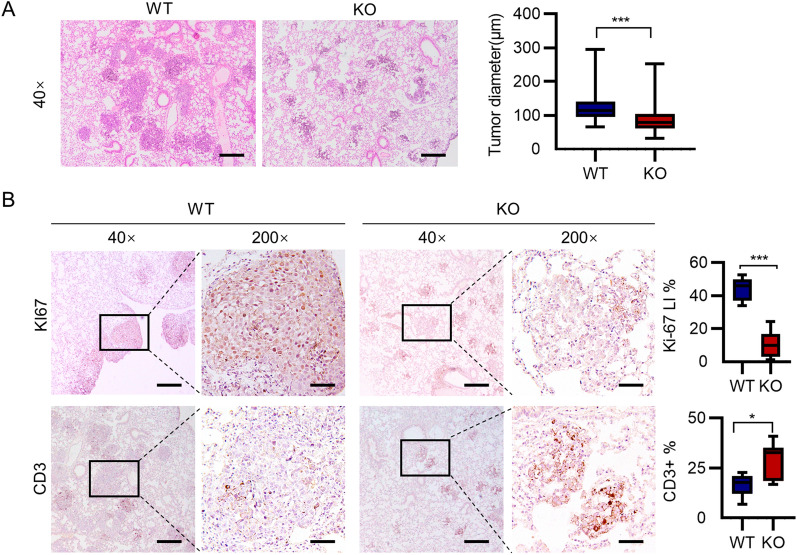
NOS1 deletion inhibits lung metastasis of melanoma. ** A** HE staining shows the diameter of lung nodule was small in NOS1-KO group than WT (***$\hbox {p} < 0.001$). ** B** Immunohistochemistry shows the intensity of Ki67 were decreased and CD3 increased in NOS1-KO than WT group. The proliferation index of Ki-67 was investigated through IHC in xenograft tumors. Scale bar: $50 \; \upmu \hbox {m}$

To validate the role of NOS1 in immune escape, we established lung metastasis of melanoma cells with or without NOS1 expression (NOS1-KO B16 and WT B16) in immune competent C57BL/6 mouse. MTT assay indicated consistently that the cell proliferation of NOS1-KO B16 was slower than WT B16 ($\hbox {p} < 0.001$, Additional file 3: Fig. S2). $5\times 10{}^5$ cells harvested at logarithm stage in culture were injected into C57BL/6 mice through tail vein for generating lung metastasis model ($\hbox {n}=7$). Ten days after injection, mice were sacrificed humanely and the number and size of metastatic nodules were evaluated. As shown in Fig. 4, the size of tumor nodes were significantly smaller in NOS1-KO groups ($\hbox {p}<0.001$, Fig. 4A).

The histopathology with hematoxylin and eosin (H&E) staining showed that nodules of NOS1-KO melanoma have less number of melanoma cells and display poor cell morphology compared with the WT group. Ki67 staining also showed that proliferating cells in NOS1-KO nudes were significantly less than control groups ($\hbox {p}<0.001$, Fig. 4B). The immune cell infiltration in lung nodules were detected by staining with the pan-T cell marker CD3 ($\hbox {p}<0.05$, Fig. 4B). The percentages of CD3 positive cells in NOS1-KO nodules were significantly higher than the control group (Fig. 4B). The results confirmed that NOS1 knockout inhibited melanoma growth of lung metastasis and increased the immune cell infiltration. However, the immune inhibitory role modification by NOS1 expression might also contribute to the involve in its growth promotion in of melanoma.

### The NOS1 regulated immune response is correlated with clinical outcome

**Fig. 5:**
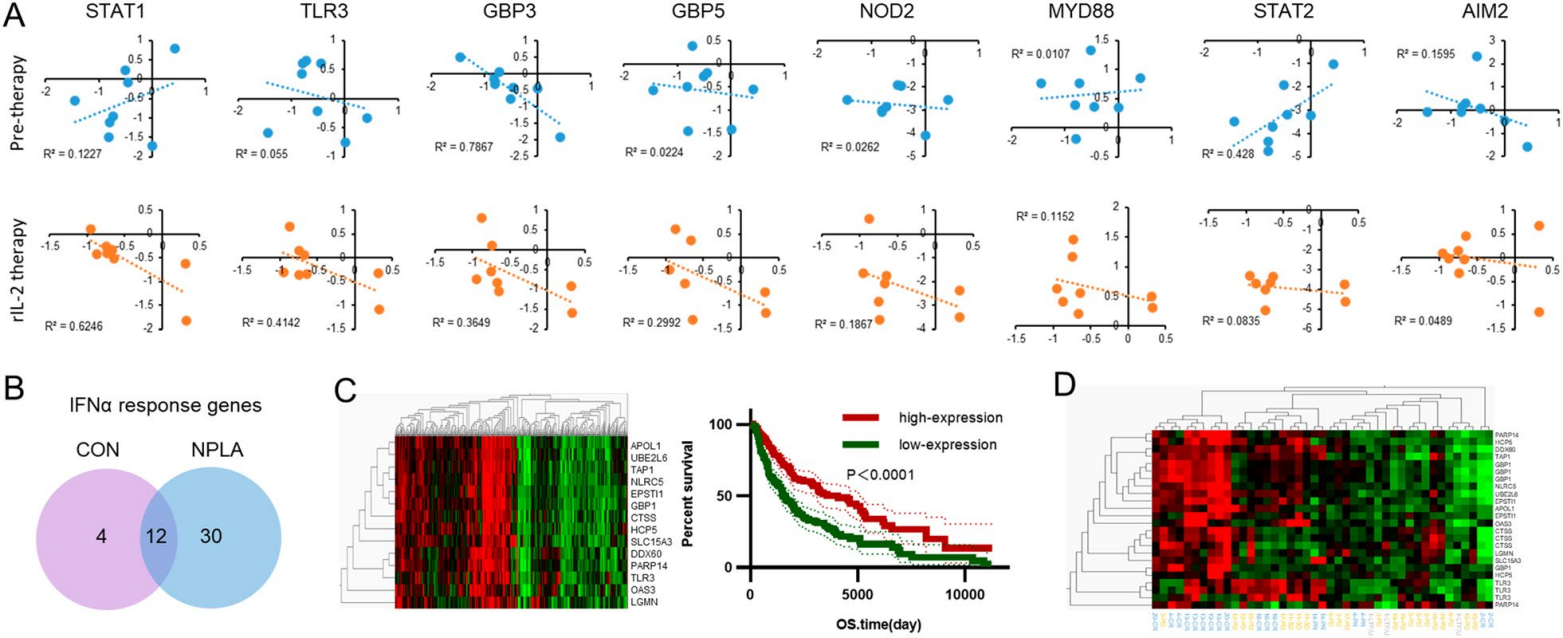
NOS1-inhibition increase the clinical effect of melanoma. ** A**STAT1, TLR3, GBP3 and GBP5 significantly negatively correlated with NOS1. $R{}^2>0.25$. ** B** The graph of NPLA-treatment group (IFN$\upalpha$-NPLA vs. NPLA) and control group (IFN$\upalpha$ vs. control) showed 30 unique NOS1-IFN related genes. ** C** Left panel: The heatmap of 14 NOS1-IFN related genes in 471 clinical samples. Right panel: The overall survival time of the group of high-expression NOS1-IFN related genes was significantly longer than that of the low expression group. (***$\hbox {p} < 0.001$). ** D** The remission of the high-expression NOS1-IFN related genes group was significantly longer than that of the low expression group. (**$\hbox {p} < 0.01$)

To better understand the role of NOS1 expression on immune modulation and clinical significance in melanoma, we investigated a cohort of transcription data of melanoma under high dose of rIL2 treatment down load from GEO (GSE32611). We analyzed the correlation of NOS1 expression with the key components (TLR3, MYD88, STAT1/2, OAS1, NOD2 and GBP3/5) in immune response pathways, JAK-STAT, NOD-LIKE, TOLL-LIKE pathway. Before rIL2 treatment, only GBP3 was negatively correlated with NOS1 expression (Fig. 5A). However, expression of eight genes encoding key immune factors were all negatively correlated with NOS1 after immune therapy (TLR3, STAT1, and GBP3/5 with $R{}^2> 0.25$; Fig. 5A). Consistent with our transcription data in vitro, NOS1 expression is correlated negatively with the function of immunomodulation pathways.

We further explored the clinical significance of NOS1 expression and its immune modulation from TCGA database of melanoma patients. Of 471 melanoma clinical samples, NOS1 expressed low level in most samples and found no different among four clinical stages (Additional file 4: Fig.S3) and could not predicate the survival prognosis of patients (data not shown). With stringent cut off (p-adjust $< 0.05$), 16 ISGs were identified in A375 (con-ISGs), while 42 ISGs identified in the NPLA-treatment group (NPLA -ISGs). Venn diagram classed 30 genes uniquely in NOS1 inhibited group, which defined as NOS1 modulated immune response gene (NOS1-ISGs) (Fig. 5B). Immune gene signatures have been shown to be associated with patient outcomes such as disease-free and overall survival, response to chemotherapy. We downloaded 471 transcriptional profiles associated with their clinical parameters. Through hierarchical clustering analysis (Qlucore Omics Explorer 3.2), 14 NOS1-ISGs self-organize 471 patients into major two groups, high or low NOS1-ISGs groups. Examined the prognostic performance of the two groups by Kaplan–Meier analysis showed that the overall survival time of the high-NOS1-ISGs group was significantly longer than that of the low -NOS1-ISGs group ($\hbox {p}<0.001$, Fig. 5C). We further analyzed the tumor remission by using these 14 NOS1-ISGs in 19 patients under chemotherapy (GSE10282). As showed in Fig. 5D that the tumor remission in high-NOS1-ISGs group was more significant than low-NOS1-ISGs group.

## Discussion

Nitric oxide is a soluble endogenous gas working as a critical metabolic regulator in metabolism reprogramming of tumorgenesis [25]. NO-induced S-nitrosation activates oncogenic signaling cascades or directly alters activities of metabolic enzymes [26]. Our previous study showed that NOS1 expression in ovarian cancer promoted tumor glycolysis and growth through S-nitrasylation of the key enzyme (PFK1) [20]. In this study, we also observed that metabolic reprogramming is the major alteration in NOS1 deleted melanoma cells. NOS1 high-expression accompanies with up-regulation of multiple metabolic pathways for macromolecule synthesis and down-regulation of mitochondrial respiration [27, 28]. Thus, NOS1 expression might shift metabolism of redirect nutrients into anabolic pathways to maintain biomass production for supporting uncontrolled proliferation of cancer cells. Our data showed that NOS1 upregulated the regeneration of arginine. It has been indicated that arginine is a strong inducer of histone acetylation and deprivation of arginine leads to high histone methylation such as H3K9me3 and H3K27me3 [29], contributing the silencing of genes involved in mitochondrial functions including OXPHOS, purine and pyrimidine synthesis, DNA repair, etc [30–32]. Whether regulation of the arginine regeneration is a causal mechanism for NOS1 related global enhanced expression of metabolic, mitochondrial and pyrimidine synthesis needs to be further studied.

Innate immunity is crucial for immune-surveillance and anti-tumor therapy [33, 34]. NO generated by tumor cells impairs the TLR signaling pathway in anti-tumor immunity [21]. In this study, NOS1 deletion in melanoma up-regulated the expression of IFN$\upalpha$ regulating genes. NOS1 related immune response genes prognosis poor chemotherapy efficacy and shorter overall survival of melanoma patients. It is noticed that NOS1 induced a significant alteration of transcription profiles only under condition of IFN$\upalpha$ stimulation but not in basal levels, indicating that NOS1 impaired the activity of immune regulatory signaling. Most NOS1-repressing interferon-response genes are cytokines and chemokines downstream of the JAK-STAT and TOLL–LIKE signaling pathways. The releases of these molecules play a critical role in the activation of interferon-induced antitumor immune-response. It was confirmed by the observation of the increased recruitment of CD3+ cells in NOS1-deleted tumors. Deletion of NOS1 leads to down-regulation of innate immune and switching “cold” tumors to “hot”, suggesting NOS1 could be a potential target for tumor immune therapy.

Arginine metabolism has recently emerged as a critical pathway for controlling immune cell function [35]. In addition to epigenetical regulation of gene transcription, arginine is a precursor for polyamine synthesis [36]. In cancer cells, polyamine metabolism is frequently dysregulated, which promotes transformation and tumor progression [37, 38]. Inhibition of polyamine synthesis induces autonomous cancer cell death and enhances immune response [37, 39]. However, the causal relationship between polyamine synthesis and NOS1-induced downregulation of innate immune signaling needs to be further studied.

## Conclusions

Our finding indicated that NOS1 plays a critical role in immune escape of melanoma. The immunogenic heterogeneity of human melanoma could be classified by NOS1 regulated immune response genes. NOS1 signature predicts chemotherapy efficacy and the survival prognosis for melanoma patients. Targeting NOS1 might be benefit in melanoma therapeutic management.

## Supplementary Information

**Additional file 1: Fig: S1.** The sequence analysis of recombinant plasmid p2u6-NOS1.

**Additional file 2: Table S1.** GSEA result of the transcriptome data.

**Additional file 3: Fig. S2.** MTT assay showed NOS1-KO B16 cells grow slower than WT group (***p < 0.001).

**Additional file 4: Fig. S3.** NOS1 expression level among four clinical stages in 471 melanoma clinical samples.

## Data Availability

The datasets generated and/or analyzed during the current study are available from the corresponding author on reasonable request.

## Abbreviations

NOS1: Nitric Oxide Synthase 1
NO: Nitric oxide
GSEA: Gene set enrichment analysis
DEGs: Differential expression genes
IFN: Interferon
ISGs: Interferon-stimulated genes
ODC: Ornithine decarboxylase
SAT1: Spermidine/spermine-N1-acetyltransferase
ASS1: Argininosuccinate synthase 1
