# Supplementary figures and images for: Inhibition of NOS1 promotes the interferon response of melanoma cells
Source: J Transl Med. 2022 May 10;20:205. doi: 10.1186/s12967-022-03403-w (PMC9092760; doi:10.1186/s12967-022-03403-w)

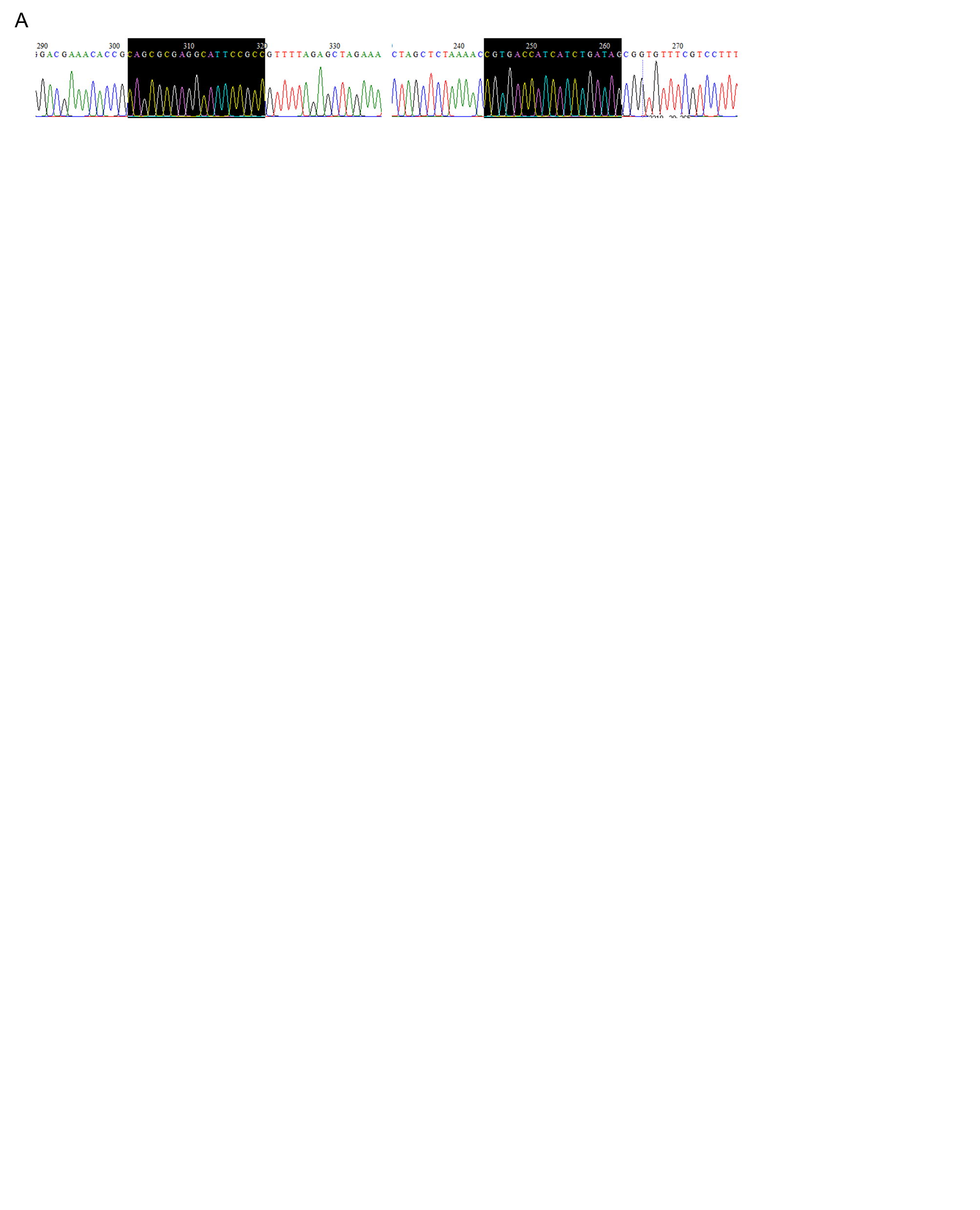

Supplement: Supplementary file 1 — Additional file 1: Fig: S1. The sequence analysis of recombinant plasmid p2u6-NOS1. [file 12967_2022_3403_MOESM1_ESM.tif]

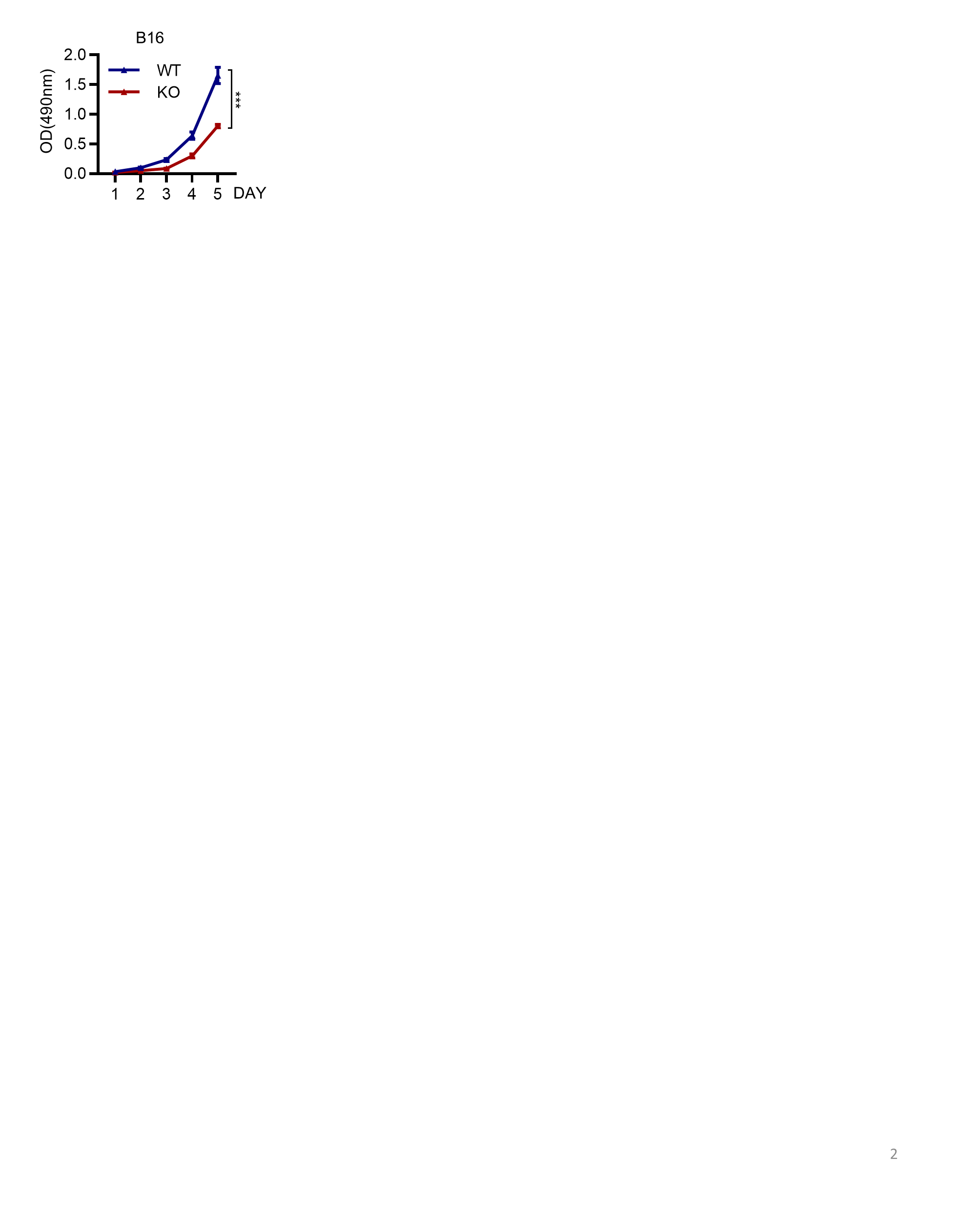

Supplement: Supplementary file 3 — Additional file 3: Fig. S2. MTT assay showed NOS1-KO B16 cells grow slower than WT group (***p < 0.001). [file 12967_2022_3403_MOESM3_ESM.tif]

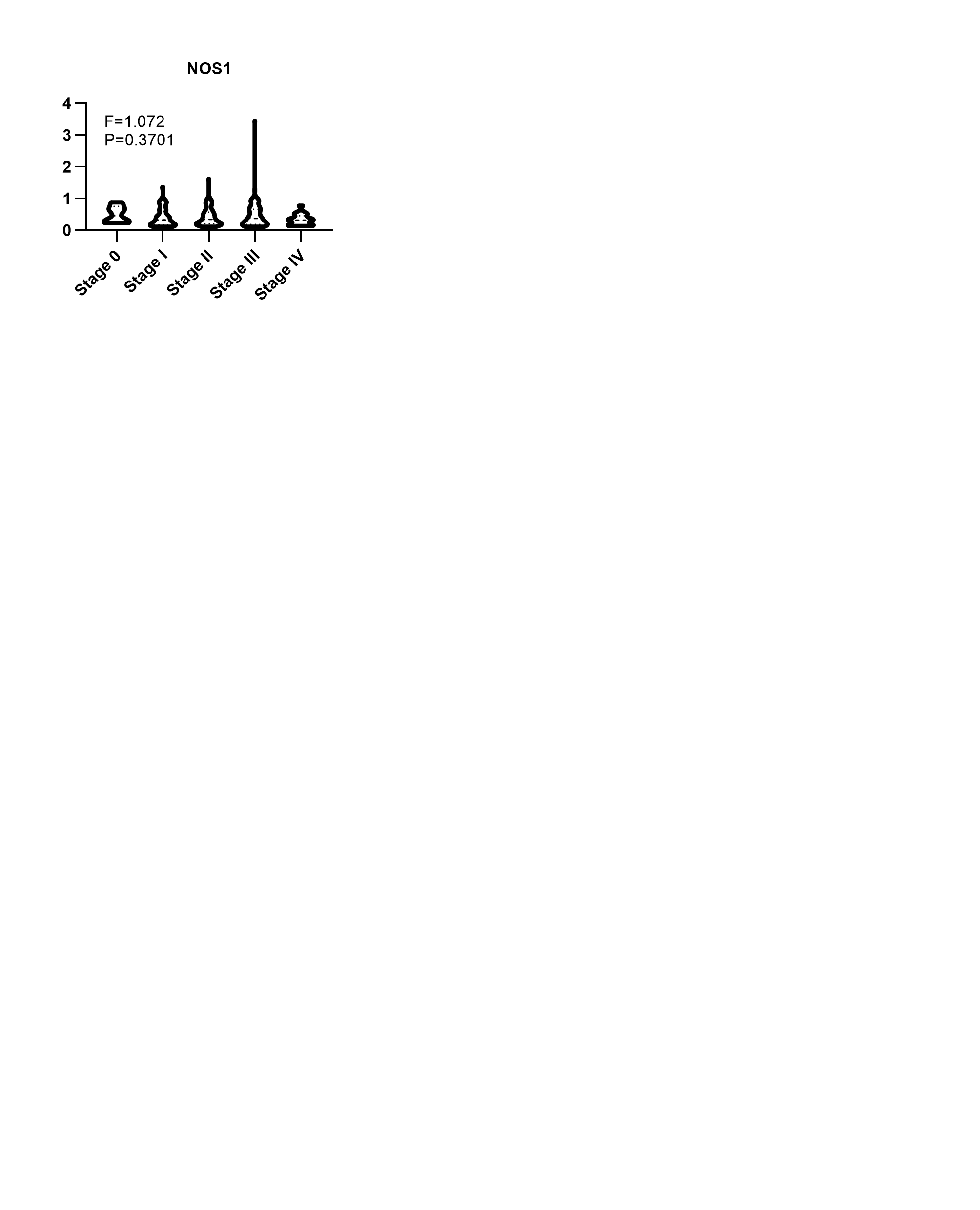

Supplement: Supplementary file 4 — Additional file 4: Fig. S3. NOS1 expression level among four clinical stages in 471 melanoma clinical samples. [file 12967_2022_3403_MOESM4_ESM.tif]
